# Novel mutations in the *marR* gene (*MAB_2648c*) modify nitroxoline activity in *Mycobacterium abscessus*

**DOI:** 10.1128/aac.01744-24

**Published:** 2025-04-01

**Authors:** Pusheng Xu, Yi Li, Bihan Xu, Dan Cao, Xu Dong, Yanghui Xiang, Xiuzhi Jiang, Xin Yuan, Yuwei Qiu, Ying Zhang

**Affiliations:** 1 State Key Laboratory for Diagnosis and Treatment of Infectious Diseases, National Clinical Research Center for Infectious Diseases, The First Affiliated Hospital, Zhejiang University School of Medicine 71069, Hangzhou, Zhejiang, China; 2 Jinan Microecological Biomedicine Shandong Laboratory 661980 , Jinan, Shandong, China; St. George's, University of London, London, United Kingdom

**Keywords:** *Mycobacterium abscessus*, antibiotic resistance, mutant strains, nitroxoline

## Abstract

*Mycobacterium abscessus* is a fast-growing nontuberculous mycobacterium that can cause severe disease and poses a significant challenge for clinical management. New drugs are urgently needed for more effective treatment. Nitroxoline, an antibiotic used for treating urinary tract infections in Europe, has recently been shown to have promising activity against *M. abscessus*. However, the mechanism of its modified activity is not known. In anticipation for its potential clinical use, in this study, we investigated the mechanism of nitroxoline activity modification through mutant selection followed by whole-genome sequencing. We identified mutations in a transcriptional repressor MarR (*MAB_2648*c) controlling the expression of efflux pumps MmpS5-MmpL5 family that are associated with nitroxoline activity modification. Complementation of the mutants with the wild-type *marR* gene restored the MIC values to levels comparable to those of the wild-type strain. This study elucidates the key mechanism of how *M. abscessus* mutations modify the activity of nitroxoline, which has significant implications for rapid molecular detection of drug-resistant *M. abscessus* and for further studies on resistance mechanisms.

## INTRODUCTION

The *Mycobacterium abscessus* complex (MABC) has become an increasingly severe public health challenge due to its rapid growth, multidrug resistance, and ability to cause a variety of infections in humans. The MABC subspecies, including *M. abscessus*, *M. massiliense*, and *M. bolletii*, primarily affect the skin, soft tissues, and lungs but can also cause infections in other sites such as the central nervous system, bloodstream, and eyes. In MABC, *M. abscessus* (the *M. abscessus* mentioned in this article refers to *M. abscessus* subsp. *abscessus*) is the most common pathogen, and the rapid development of its drug resistance makes treatment difficult. Mechanisms of resistance include mutation of the drug target, low permeability of the cell wall, induction of drug efflux pumps, and inactivation of activating enzymes (1, 2). For example, clarithromycin and azithromycin are cornerstone drugs for the treatment of *M. abscessus*. These drugs can induce the expression of the *erm(41*) gene, which encodes a ribosomal methyltransferase that methylates the A2058 nucleotide of 23S ribosomal RNA (rRNA), thereby leading to macrolide resistance (3). The *erm(41*) gene varies among subspecies with *M. massiliense* containing a deletion in *erm(41*), leading to different resistance profiles and treatment outcomes with macrolide (4, 5). Efflux-related mechanisms are also significant contributors to resistance. Mutations in TetR family transcriptional repressor MarR type genes, such as *MAB_2299*c, lead to increased expression of MmpS-MmpL efflux pump, resulting in resistance to clofazimine and bedaquiline in *M. abscessus* (6, 7). In addition, mutations in a different TetR family transcriptional repressor MarR (*MAB_2648*c) have been shown to confer specific resistance to ethionamide (ETH) by regulating the expression of the MmpS5-MmpL5 efflux pump system (8). Likewise, *MAB_4384* operates through an analogous mechanism to MarR, where its mutations result in increased expression of MmpS5-MmpL5 causing resistance to thiacetazone analogs (9). Therefore*,* it is important to study the effect of drug-resistance genes on treatment of *M. abscessus* infections (10).

Nitroxoline is a promising class of compounds known for its antibacterial activity against uropathogenic *Escherichia coli*. It is a clinically used drug in Europe for treating urinary tract infections (11, 12). Recently, Hoffmann et al. (13) have shown that nitroxoline had good activity (MIC = 2–4 µg/mL) against *M*. *abscessus in vitro*. However, its mechanisms of action and resistance in *M*. *abscessus* are unknown. In *E. coli*, mutations in the transcriptional repressor EmrR have been found to confer low-level nitroxoline resistance by upregulating the EmrAB-TolC efflux pump, while higher-level resistance involves additional mutations that increase *tolC* expression (14). In anticipation for its potential treatment of *M*. *abscessus* infections, here, we isolated 18 mutant strains of *M. abscessus* with modified nitroxoline activity *and subjected* them to whole-genome sequencing to identify possible mutations involved in reduced nitroxoline activity. We found novel mutations in *marR* encoding a potential transcription repressor for efflux pump MmpS5-MmpL5, which is not previously reported, to be associated with reduced nitroxoline activity. Introducing a wild-type copy of the *marR* gene into the mutant strain restored the MIC values to levels comparable to those of the wild-type strain*,* which confirms the role of *marR* mutation in modifying nitroxoline activity.

## RESULTS

### Nitroxoline MIC for *M. abscessus* ATCC 19977

Using the broth microdilution test, we determined the MIC of nitroxoline against *M. abscessus* as 2–4 μg/mL. This result indicates that nitroxoline possesses good inhibitory activity against *M. abscessus* at relatively low concentrations, underscoring its potential as a candidate for further investigation in treating infections caused by this pathogen.

### Isolation of *M. abscessus* mutant strains with reduced nitroxoline susceptibility

We prepared nitroxoline-containing plates at 2 × MIC*,* 4 × MIC*,* 8 × MIC*,* 16 × MIC, and 32 × MIC (“×” means “times”). Then we applied stationary phase culture of *M. abscessus* to the plates and found growth at 2 × MIC*,* 4 × MIC but no growth on plates containing 8 × MIC*,* 16 × MIC and 32 × MIC. So, we plated *M. abscessus* on 20 nitroxoline-containing plates at 16 × MIC and 32 × MIC, respectively, to obtain a sufficient number of mutants for analysis. However, no growth of bacteria was found on plates at 32 × MIC*,* but from plates at 16 × MIC*,* 18 mutant colonies were identified and re-plated on the newly prepared plates containing 16 × MIC of nitroxoline. Based on the number of cells plated and the mutants obtained, the mutation frequency of resistant mutants at 32 µg/mL of nitroxoline was approximately 1 × 10⁻^8^. The mutants were then subjected to whole-genome sequencing (see below).

### Whole-genome sequencing identified *marR* mutations associated with reduced nitroxoline susceptibility in *M. abscessus*

**Fig 1:**
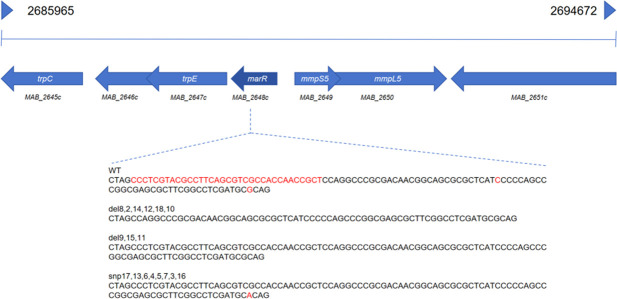
Upstream and downstream genes and mutation sites of *marR (MAB_2648c*). The nucleotides highlighted in red indicate mutation sites in mutant strains with reduced nitroxoline susceptibility. Mutant strains 2, 8, 10, 12, 14, and 18 had a 34 bp deletion mutation (CCCTCGTACGCCTTCAGCGTCGCCACCAACCGCT) at nucleotide position 455–488. Mutant strains 9, 11, and 15 exhibited a single nucleotide C deletion at nucleotide position 424. Mutant strains 3, 4, 5, 6, 7, 13, 16, and 17 had a point mutation (G→A) at nucleotide position 391 causing amino acid substitution of Arg131Cys.

Whole-genome sequencing analysis showed that 17 of the 18 mutant strains had deletion mutations or point mutations only in the *marR* gene compared to the parent strain ATCC19977. Then Sanger sequencing was performed on PCR products containing the *marR* gene from the mutant strains and confirmed six of the mutants had a 34 bp deletion of nucleotides from 455 to 488 (CCCTCGTACGCCTTCAGCGTCGCCACCAACCGCT) of the *marR* gene (*MAB_2648*c) and three were cytosine deletion at nucleotide position 424. The point mutation of G-to-A change was located at nucleotide position 391 (Table 1; Fig. 1). The deletion mutations would result in the early termination of the protein product MarR*,* and the point mutation G-to-A change at nucleotide position 391 would cause a change of amino acid from Arginine to Cysteine at 131 of the MarR protein. MarR represents a subtype of TetR transcriptional repressors*,* many of which are associated with resistance of different bacteria to functionally different antibiotics (15). We selected two representative mutant strains 15 and 16 for further studies. Mutant strain 15 has a deletion mutation at nucleotide position 424 and the mutant strain 16 has point mutation at nucleotide position 391, causing amino acid substitution of Arg131Cys. We tested the MICs of both mutant strains and the parent strain to nitroxoline*,* as well as its derivatives (5-Nitroso-8-hydroxyquinoline; 5,7-Dibromo-8-hydroxyquinoline) and several other antibiotics (clarithromycin, amikacin, rifabutin, sitafloxacin, bedaquiline, clofazimine) commonly used against *M. abscessus* and found that the mutant strains are specific for nitroxoline activity modification.

**TABLE 1:** Mutations in *MAB_2648*c (*marR*) identified from *M. abscessus* mutants with reduced nitroxoline susceptibility by whole-genome sequencing

Strains	Nucleotide mutation	Frequency (%)	Count	Effect
8, 2, 14, 12, 18, 10	Deletion of CCCTCGTACGCCTTCAGCGTCGCCACCAACCGCT (455_488)	32	6	Frameshift mutation
9, 15, 11	Deletion of C (424)	16	3	Frameshift mutation
17, 13, 6, 4, 5, 7, 3, 16	SNP of G to A (391)	42	8	Missense mutation Arg131Cys

### *marR* mutant strains with reduced nitroxoline susceptibility show restored MIC levels after complementation with the *marR* gene

To determine if the identified mutations in the *marR* gene are responsible for the modified nitroxoline activity*,* we subsequently introduced the wild-type *marR* gene into the spontaneous mutants and found that the MIC values of the mutants returned to levels comparable to those of the wild-type strain (Table 3). This result demonstrates that the *marR* mutation is indeed responsible for the observed modification of nitroxoline activity, as restoring the wild-type *marR* gene re-establishes the suppression of efflux pump expression*,* thereby reducing nitroxoline resistance (9).

This complementation experiment provides compelling evidence that MarR is a repressor of drug efflux in *M. abscessus*. When *marR* is mutated*,* the repressive function is lost*,* leading to unregulated efflux pump expression and consequently higher resistance levels (16). Reintroduction of the wild-type *marR* re-establishes its regulatory control*,* reducing efflux activity and allowing nitroxoline to accumulate intracellularly*,* thereby restoring its antibacterial efficacy (17).

## DISCUSSION

In this study, we identify the *marR* gene (*MAB_2648*c) as a critical factor in modulating nitroxoline activity in *M. abscessus*. Through whole-genome sequencing and MIC testing, we found that mutations in *marR*, including both deletions and point mutations, are associated with reduced nitroxoline activity in mutant strains compared to the wild-type strain (Table 1). Importantly, complementation of the mutant strains with the wild-type *marR* gene restored the MIC values to levels comparable to those of the wild-type strain, confirming *marR*’s role in regulating nitroxoline activity (Table 3). Our findings advance the understanding of nitroxoline’s mechanism of action and resistance and its potential applications. Specifically, this study demonstrates that mutations in the *marR* gene (*MAB_2648*c) play a critical role in modulating nitroxoline activity in *M. abscessus*. In addition, our study expands upon existing research, by showing that mutations in *marR* confer resistance to not only nitroxoline but also ethionamide, but not other drugs (Table 2). Our findings that *marR* mutations are the primary mechanism of resistance to nitroxoline provide the molecular basis for developing molecular tests for rapid detection of nitroxoline resistance, in anticipation for its clinical use in the future based on its promising activity against *M. abscessus*.

**TABLE 2:** MICs of *M. abscessus* wild-type strain and mutant strains with reduced nitroxoline susceptibility

Drug	ATCC 19977 (μg/mL）	Mutant strain 15 (μg/mL）	Mutant strain 16 (μg/mL）
Nitroxoline	2	16	16
Clarithromycin	8	8	8
Amikacin	4	4	4
Bedaquiline	<0.06	<0.06	<0.06
Clofazimine	0.5	0.5	0.5
Rifabutin	8	8	8
Sitafloxacin	4	4	4
Metronidazole	>128	>128	>128
Delamanid	>128	>128	>128
Ethionamide	64	128	128
5-Nitroso-8-hydroxyquinoline	>128	>128	>128
5,7-Dibromo-8-hydroxyquinoline	>128	>128	>128

The *marR* gene (*MAB_2648*c), which encodes a transcriptional regulator that represses MmpS5/MmpL5 efflux pump expression, has previously been identified as a contributor to ethionamide (ETH) resistance in *M. abscessus* (8). Our study extends the above finding by identifying the same gene (*MAB_2648*c) being involved in modulating nitroxoline activity, revealing a relatively narrow specificity of MarR in conferring reduced susceptibility to specific drugs. Furthermore, our findings indicate that mutants with reduced nitroxoline susceptibility do not exhibit cross-resistance to many other tested drugs such as clarithromycin, amikacin, rifamycin, fluoroquinolone, linezolid, clofazimine and bedaquiline, except for ETH (Table 2 and 3), suggesting a relative specificity. This is similar to previous findings with *M. abscessus MAB_2299*c *and M. tuberculosis Rv0678* (MmpR) where their mutations resulted in cross-resistance to both clofazimine and bedaquiline due to the upregulation of the MmpS5/MmpL5 efflux pump (18, 19). These findings suggest that different MarRs in *M. abscessus* control different efflux pumps and exhibit relatively narrow substrate specificity depending on the antibiotic class, underscoring the complexity of efflux-mediated resistance in this organism.

**TABLE 3:** MICs of *M. abscessus marR* (*MAB_2648*c) mutant strains with reduced nitroxoline susceptibility after complementation with the wild-type gene

Drug	ATCC 19977 (μg/mL)	Mutant strain 15 + (pMV306hsp vector alone) (μg/mL)	Mutant strain 15 + (pMV306hspWT*marR*) (μg/ml)	Mutant strain 16 + (pMV306hsp vector alone) (μg/mL)	Mutant strain 16 + (pMV306hspWT*marR*) (μg/mL)
Nitroxoline	2	16	4	16	4
Clarithromycin	8	8	8	8	8
Amikacin	4	4	4	4	4
Bedaquiline	<0.06	<0.06	<0.06	<0.06	<0.06
Clofazimine	0.5	0.5	0.5	0.5	0.5
Rifabutin	8	8	8	8	8
Sitafloxacin	4	4	4	4	4
Ethionamide	64	128	64	128	64

MarR proteins are transcriptional repressors that control the expression of multidrug efflux pumps, playing a crucial role in bacterial antibiotic resistance. These proteins function as homodimers with a DNA-binding domain that interacts with specific DNA sequences, and a ligand-binding domain that induces conformational changes upon ligand binding. These changes reduce the proteins’ DNA-binding affinity, leading to the derepression of efflux pump genes and increased antibiotic resistance (20). Interestingly, these mutations are associated with reduced susceptibility to both nitroxoline and ETH, despite the structural differences between the two drugs. This raises the question of how a single *marR* mutation can mediate activity modification to two distinct compounds. One possible explanation is that mutations in *marR* result in the upregulation of the MmpS5-MmpL5 efflux pump system, which may export nitroxoline and ETH as substrates but not other antibiotics, such as clarithromycin or amikacin, suggesting a degree of substrate specificity in the MmpS5-MmpL5 efflux pump. This specificity raises important questions about the molecular basis of substrate recognition and exclusion within the pump. Further investigations into the molecular details of MarR-mediated efflux regulation and the substrate specificity of the MmpL5-MmpS5 efflux pump using structural biology tools will be valuable for understanding activity modification patterns and could inform the design of novel inhibitors to counteract drug-resistant *M. abscessus* strains.

Nitroxoline has traditionally been used for the treatment of urinary tract infections due to its high concentration in urine. However, nitroxoline and its analogs have shown promising antibacterial activity against mycobacteria, including *M. tuberculosis* (21) and *M. abscessus* (13). For example, Shah et al. demonstrated that the structurally related compound 8-hydroxyquinoline exhibits a bactericidal effect against *M. tuberculosis* and kills the bacterium within macrophages (21). Similarly, nitroxoline exhibits excellent activity against multidrug-resistant *M. tuberculosis* isolates (22). Our findings that the MIC (2–4 µg/mL) of nitroxoline for *M. abscessus* is lower than its *C*_max_ range 5.4–9.5 µg/mL (23) (see Table 2) have implications for its effectiveness in treating *M. abscessus* infections beyond its traditional use in urinary tract infections. Future studies utilizing macrophage models and animal models will be required to evaluate the efficacy of nitroxoline against *M. abscessus* infection *in vivo*.

There are some limitations to this study. The number of mutant strains screened was relatively small, which may limit the generalizability of our findings. It is important to note that the MIC values reported in our study are derived from *in vitro* experiments from a limited number of *M. abscessus* strains and reflect microbiological activity patterns under controlled laboratory conditions. These values do not directly correlate with clinical susceptibility or resistance, as *in vivo* factors such as drug pharmacokinetics and pharmacodynamics were not addressed in our study. While our findings demonstrate that mutations in the *marR* gene can modify the activity of nitroxoline in *M. abscessus*, the current study design does not allow us to determine whether these mutations could stratify isolates into clinically relevant “susceptible” or “resistant” categories. According to EUCAST-defined breakpoints (Clinical breakpoints v 15.0), an MIC value higher than 16 µg/mL is considered resistant to nitroxoline for uropathogenic *E. coli* (24). In the case of *M. abscessus,* clinical susceptibility or resistance for nitroxoline remains to be determined. Additionally, functional validation of *marR*’s role in activity modification was not comprehensive, leaving room for further detailed investigation. The specific target of nitroxoline remains unknown and should be addressed in future studies to fully understand its mechanisms of action and to identify any new resistance mechanisms beyond *marR* mutations.

In summary, our study establishes *marR* (*MAB_2648*c) as a primary mechanism underlying nitroxoline activity modification in *M. abscessus*. This finding highlights the importance of *marR* in understanding resistance mechanisms, as mutations in this gene can significantly impact the regulation of efflux pumps, contributing to changes in activity not only to nitroxoline but also to ethionamide. However, the specific target of nitroxoline within *M. abscessus* remains unknown, underscoring the need for future research to uncover the precise molecular interactions involved. Identifying these targets could lead to an improved understanding of mechanisms of nitroxoline resistance and pave the way for developing more effective drugs against drug-resistant *M. abscessus* infections. In addition, future research should evaluate nitroxoline’s *in vivo* efficacy and explore its potential in combination therapies or other clinical applications for more effective treatment of *M. abscessus* infections. Identifying the resistance mechanisms could pave the way for developing rapid molecular tests for the detection of resistance and more effective strategies against drug-resistant mycobacterial infections.

## MATERIALS AND METHODS

### Bacterial growth and culture conditions

*M. abscessus* strain ATCC 19977 was grown in Middlebrook 7H9 medium (Becton Dickinson) supplemented with oleic acid-dextrose-catalase (OADC*,* Aladdin) and 0.05% (vol/vol) Tween 80 (Sigma-Aldrich). For growth on solid media*, M. abscessus* was cultured on Middlebrook 7H11 plates with OADC. Kanamycin (MCE) at 50 µg/mL was supplemented when appropriate for *M. abscessus*. *E. coli* was grown in LB broth or LB agar supplemented with 50 µg/mL kanamycin where appropriate.

### Determination of MICs

CAMHB broth (Hopebio) was used to prepare twofold dilutions of Nitroxoline, Amikacin, Rifabutin, Sitafloxacin (Macklin); Clarithromycin, Clofazimine, Metronidazole, Delamanid, Ethionamide, 5-Nitroso-8-hydroxyquinoline, 5,7-Dibromo-8-hydroxyquinoline (Aladdin); Bedaquiline (WuXi AppTec), ranging from 128 μg/mL to 0.0625 μg/mL. Except for Amikacin, which was dissolved in ddH2O, all other compounds were dissolved in DMSO. The stationary phase cultures were diluted to about 10^6^ CFU/mL, added to antibiotic-containing 96-well plate, which was wrapped with tin foil and placed in a 37°C incubator and visible bacterial growth was observed after 5 days.

### Selection of nitroxoline resistant mutants and whole-genome sequencing

To screen for spontaneous resistant mutants, 100 µL of stationary phase *M. abscessus* ATCC19977 (1 × 10^9^ CFU) was spread on 7H11 agar plates containing 16 × and 32 × MIC, respectively. Spontaneous mutants were picked out and re-plated and amplified on plates containing nitroxoline and were subcultured in 7H9 liquid medium for 3 days. After centrifugation of 2 mL of the bacterial culture, 500 µL of TE buffer was added to the pellet, which was then inactivated in a water bath at 80°C for half an hour and subjected to whole-genome sequencing. Sequence reads were mapped to the *M. abscessus* ATCC 19977 reference genome. The whole-genome sequence data for the mutant strains were submitted to NCBI database with BioProject number PRJNA1209868.

### PCR and Sanger sequencing

To verify the mutations identified by whole-genome sequencing, the mutants of *M. abscessus* were subjected to PCR amplification using *MAB_2648*c primers (Table S1). PCR amplification parameters were as follows: heat denaturation at 98°C for 30 min followed by 30 cycles of 98°C for 10 s, 60°C for 5 s, and 72°C for 5 s and then extension at 72°C for 1 min. In the gene complementation experiment, we used pMV306hsp primers (Table S1) to verify the complementation experiment was successful. The PCR parameters were as follows: heat denaturation at 98°C for 30 min followed by 30 cycles of 98°C for 10 s, 66°C for 5 s, and 72°C for 5 s and then extension at 72°C for 1 min. The PCR products were analyzed on agarose gel by electrophoresis as described (6).

### Gene complementation experiment

The genomic DNA of *M. abscessus* ATCC 19977 was extracted using FastPure Bacteria DNA Isolation Mini Kit (Vazyme) and the target gene *marR* was amplified by PCR using appropriate primers (Table S1) followed by Fastpure Gel DNA Extraction Mini Kit (Vazyme). The pMV306hsp plasmid (8) was cleaved with *Hind*III and *Age*I, followed by cloning of the *marR* PCR product into this plasmid vector using ClonExpress Ultra One Step Cloning Kit (Vazyme). The recombinant plasmid was transformed into *E. coli* DH5α (Vazyme) by heat shock, and the transformant was incubated in a 37°C shaker for 3 h followed by plating onto LB agar containing 50 µg/mL kanamycin and incubated at 37°C overnight. On the next day*,* single colonies were picked and cultured in LB broth*,* containing 50 µg/mL kanamycin and subjected to Sanger sequencing to confirm that the wild-type *marR* gene was successfully cloned into pMV306hsp. The *marR* recombinant plasmid was then extracted with the Fastpure Plasmid Mini Kit (Vazyme) and electroporated (2,500 V*,* 1,000 Ω, and 25uF) into the mutant strains. The electroporated mutant strains were incubated in a 37°C shaker overnight and then plated onto 7H11plates containing 50 µg/mL kanamycin and incubated at 37°C for 3 days. The bacterial cultures were then subjected to Sanger sequencing to confirm that the wild-type *marR* gene was successfully introduced into the mutant strains and was tested for susceptibility to nitroxoline and other drugs as described above.

## SUPPLEMENTAL MATERIAL

Table S1 aac.01744-24-s0001.docx Primers used in this study.

ASM does not own the copyrights to Supplemental Material that may be linked to, or accessed through, an article. The authors have granted ASM a non-exclusive, world-wide license to publish the Supplemental Material files. Please contact the corresponding author directly for reuse.
