# Supplementary material for: Novel mutations in the marR gene (MAB_2648c) modify nitroxoline activity in Mycobacterium abscessus
Source: Antimicrob Agents Chemother. 2025 Apr 1;69(5):e01744-24. doi: 10.1128/aac.01744-24 (PMC12057340; doi:10.1128/aac.01744-24)
Supplement: Table S1 — Primers used in this study. [file aac.01744-24-s0001.docx]

**Table S1. Primers used in this study**

| Primer name | Sequence (5'→3') |
| --- | --- |
| *MAB_2648c*F | AAGAAGGTACCCGGACGGTT |
| *MAB_2648c*R | CCTCGTCTCACGTCGTGATC |
| pMV306hspF | ACGTTGGCACTCGCGACCGGTTGCTTGCCGAGCGGGGTG |
| pMV306hspR | TACGTCGACATCGATAAGCTTGACGCCATTCTCCCAGACCG |
